# A causal relationship between educational attainment and risk of infectious diseases: A Mendelian randomisation study

**DOI:** 10.7189/jogh.14.04089

**Published:** 2024-04-26

**Authors:** Jueheng Liu, Jiajia Ren, Xiaoming Gao, Chuchu Zhang, Guorong Deng, Jiamei Li, Ruohan Li, Xiaochuang Wang, Gang Wang

**Affiliations:** 1 Department of Critical Care Medicine, the Second Affiliated Hospital of Xi’an Jiaotong University, Xi’an, Shaanxi, China; 2 Key Laboratory of Surgical Critical Care and Life Support, Xi’an Jiaotong University, Ministry of Education, Xi’an, Shaanxi, China

## Abstract

**Background:**

Previous observational studies have investigated the association between educational attainment and sepsis, pneumonia, and urinary tract infections (UTIs). However, their findings have been susceptible to reverse causality and confounding factors. Furthermore, no study has examined the effect of educational level on the risk of infections of the skin and subcutaneous tissue (SSTIs). Thus, we aimed to evaluate the causal relationships between educational level and the risk of four infectious diseases using Mendelian randomisation (MR) techniques.

**Methods:**

We used univariable MR analysis to investigate the causal associations between educational attainment (years of schooling (n = 766 345) and holding college or university degree (n = 334 070)) and four infectious diseases (sepsis (n = 486 484), pneumonia (n = 486 484), UTIs (n = 463 010), and SSTIs (n = 218 792)). We included genetic instrumental variables with a genome-wide significance (*P* < 5 × 10^−8^) in the study. We used inverse variance-weighted estimation in the primary analysis and explored the stability of the results using multivariable MR analysis after adjusting for smoking, alcohol consumption, and body mass index.

**Results:**

Genetically predicted years of schooling were associated with a reduced risk of sepsis (odds ratio (OR) = 0.763; 95% confidence interval (CI) = 0.668–0.870, *P* = 5.525 × 10^−5^), pneumonia (OR = 0.637; 95% CI = 0.577–0.702, *P* = 1.875 × 10^−19^), UTIs (OR = 0.995; 95% CI = 0.993–0.997, *P* = 1.229 × 10^−5^), and SSTIs (OR = 0.696; 95% CI = 0.605–0.801, *P* = 4.034 × 10^−7^). We observed consistent results for the correlation between qualifications and infectious diseases. These findings remained stable in the multivariable MR analyses.

**Conclusions:**

Our findings suggest that increased educational attainment may be causally associated with a decreased risk of sepsis, pneumonia, UTIs, and SSTIs.

Infectious diseases manifest through the invasion of pathogenic microorganisms that disrupt the normal physiological functions of the host, leading to adverse effects and dysfunction [1,2]. They include a broad spectrum of conditions and have significant implications for public health and societal economic stability.

Previous research has shown community-acquired infections such as urinary tract infections (28.6%), pneumonia (22.8%), and soft tissue infections (21.8%) to be highly prevalent [3–6], while 33.3% of patients in a multicentre prospective cohort study were diagnosed with sepsis after admission [7]. According to the 2004 World Health Report, infectious diseases rank as the second leading cause of mortality globally, causing at least 15 million deaths annually, and they also contribute significantly to the global disease burden, leading to nearly 1.5 billion total disability-adjusted life years annually [8]. Therefore, identifying risk factors that affect the transmission of infectious diseases is crucial for early prevention and reducing their overall health burden.

Recently, a large-scale meta-analysis has shown an approximate 2% decrease in all-cause mortality among adults for each additional year of education attainment they had obtained [9]. Additionally, notable disparities in mortality rates were previously observed across different socioeconomic groups during the (H1N1) 2009 influenza pandemic [10]. Consequently, we hypothesise that educational achievement, a key indicator of socioeconomic status [11], may be potentially correlated with a susceptibility to infectious diseases. Previous observational studies have found that educational level influences the development and progression of several infectious diseases [12–14]. Likewise, ecological research has identified a correlation between lower educational levels and an increased incidence of sepsis [12]. Similarly, another study based on Poisson regression models demonstrated a significant increase in mortality attributed to pneumonia in educational inequalities [13]. Furthermore, Johnson et al. [14] observed that individuals with lower educational achievements had a higher risk of urinary tract infections. Meanwhile, there is currently no research on the impact of educational level on the risk of infections of the skin and subcutaneous tissue (SSTIs). Moreover, the abovementioned findings mainly come from observational studies, making them susceptible to measurement and recall biases, among others. There is also a possibility of reverse causation in the relationships between infectious diseases and educational attainment [15]. This makes traditional epidemiological tools inadequate for establishing a causal relationship between educational attainment and the risk of multiple infectious diseases.

Mendelian randomisation (MR), a method employed in genetic epidemiology, uses single-nucleotide polymorphisms (SNPs) as instrumental variables to explore the causal relationship between an exposure factor and an outcome factor [16,17]. These genetic variants are randomly assigned aduring meiosis and are not influenced by the risk of outcome factor. In comparison with observational studies, the MR approach can attenuate the impact of confounding factors and reverse causality. Previous MR studies have indicated a significant causal association between a higher body mass index (BMI), tobacco smoking, and an increased risk of infectious diseases [18,19]. To date, studies have not attempted to demonstrate a causal relationship between educational level and the risk of multiple infectious diseases. We therefore aimed to investigate the potential causal relationship between educational attainment and the risk of sepsis, pneumonia, urinary tract infections (UTIs), and SSTIs using the MR method.

## METHODS

### Study design

By adopting the MR approach, we investigated the causal relationship between educational level and the risk of infectious diseases using publicly available data from genome-wide association studies (GWASs). Our study adheres to three critical assumptions: The genetic instrumental variables should be significantly associated with exposure; the instrumental variables should not be associated with confounding factors that may affect the relationship between exposure on the outcome; and the instrumental variables should solely influence the outcome through the exposure factor [20]. As we used publicly available de-identified data of participants from studies that had already been approved by an ethical standards committee for human experimentation, we did not require separate ethical approval for our research.

#### Genetic association data sets for educational attainment

We used data on years of schooling and qualifications (college or university degree) as indicators of educational attainment. We retrieved the former dataset from the Social Science Genetic Association Consortium database, which provides summary data for 766 345 participants [21], and the latter from the Neale Laboratory database, which includes 106 305 cases and 227 765 controls (**Table 1**).

**Table 1:** Characteristics of study data

	GWAS ID	Consortium	Sample size	Number of cases	Number of controls	Year	Population
**Outcome**							
Sepsis	ieu-b-4980	UK Biobank	486 484	11 643	474 841	2021	European
Pneumonia	ieu-b-4976	UK Biobank	486 484	22 567	463 917	2021	European
UTI	ukb-b-8814	MRC-IEU	463 010	5447	457 563	2018	European
SSTI	finn-b-L12_INFECT_SKIN	FinnGen biobank	218 792	10 343	208 449	2021	European
**Exposure**							
Years of schooling	ieu-a-1239	SSGAC	766 345	NA	NA	2018	European
Qualifications	ukb-a-397	Neale Laboratory	334 070	106 305	227 765	2017	European
**Confounding factors**							
Body mass index	ieu-b-40	GIANT	681 275	NA	NA	2018	European
Alcoholic drinks per week	ieu-b-73	GWAS and Sequencing Consortium of Alcohol and Nicotine use	335 394	NA	NA	2019	European
Ever smoked	ukb-b-20261	MRC-IEU	461 066	280 508	180 558	2018	European

GWAS – genome-wide association study, SSTI – infections of the skin and subcutaneous tissue, UTI – urinary tract infection

#### Genetic association data sets for four infectious diseases

We focussed on four infectious diseases as outcome factors – sepsis, pneumonia, UTIs, and SSTIs. We retrieved their summary-level GWAS results from the UK Biobank (sepsis and pneumonia) [22], the Medical Research Council Integrative Epidemiology Unit (UTIs) [23], and the FinnGen biobank (SSTIs) (**Table 1**). Meanwhile we derived all exposure and outcome data from the European population.

### Selection of genetic instruments

We defined specific inclusion criteria for the genetic instrumental variables (Table S1 in the **Online Supplementary Document**). First, we included SNPs with a genome-wide significance (*P* < 5 × 10^−8^) to ensure the genetic variant was genuinely associated with the exposure [24]. Then, to eliminate linkage disequilibrium, we excluded SNPs with an *r*^2^ value greater than 0.001 and located within 10 000 kb of each other. Furthermore, we used the *F*-statistic to evaluate the strength of the relationship between instrumental variables and phenotype.

Our criterion for selecting SNPs with an F-statistic greater than 10 ensures a strong correlation between genetic variants and educational attainment, thereby minimising potential instrument bias [25]. The formula for calculating *F* is given as: *F* = *R*^2^(*N* − 2)/(1 − *R^2^*), where *R*^2^ = 2 × *MAF* × (1 − *MAF*)  × *β*^2^ [26]. Here, *N* is the number of participants, *R*^2^ is the variance in exposure illustrated by the genetic variant, *MAF* is the minor allele frequency, and *β* is the effect estimate of the genetic variant in the exposure GWAS [26].

Lastly, we excluded SNPs with incompatible alleles and palindromic sequences with intermediate allele frequencies.

### Mendelian randomisation analysis

We used five methods to evaluate the causal effects of educational level on the risk of infections: MR-Egger regression, the weighted median, random-effects inverse variance weighted (IVW) approach, the simple mode, and the weighted mode. We considered the IVW approach as the primary statistical method for examining the causal relationship between educational level and the risk of infectious diseases because of its accurate estimation [27]. The remaining four approaches served as supplementary methods. Furthermore, we used the MR Steiger test to assess the directionality of the causal relationship between the exposure and the outcome [28]. As prior studies have indicated that smoking, alcohol consumption, and high BMI are risk factors for infectious diseases [19,29,30], we conducted a multivariable MR analysis to investigate this relationship.

### Sensitivity analysis

We assessed for heterogeneity in the IVW and MR-Egger results using Cochran’s Q statistic [31]. Because the *P* value of Cochran’s Q statistic was less than 0.05, indicating heterogeneity, we adopted the random-effects model of the IVW method. To address potential latent pleiotropy, we used MR pleiotropy residual (MR-PRESSO) to identify and correct for outlier SNPs [32]. In addition, we employed the MR-Egger intercept method to assess for uneven pleiotropy by testing the intercept of the MR-Egger regression as an indicator of directional pleiotropy [33]. Then, we determine whether individual SNPs had disproportionate influence on the combined estimates through the leave-one-out method [34]. Lastly, we used forest plots to estimate the effect size of each SNP on infections in relation to educational level and scatter plots to visualize the causal effects of educational level on infections. We analysed all data in R, version 4.2.2 (R Core Team, Vienna, Austria) with the ‘TwoSampleMR’ and ‘MRPRESSO’ packages. A *P*-value <0.05 denoted statistical significance.

## RESULTS

### Characteristics of the genetic instruments

Following the rigorous steps for instrumental variable selection, we identified 312 SNPs as instrumental variables for years of schooling and 186 as instrumental variables for qualifications (Table S1–9 in the **Online Supplementary Document**). The *F*-statistic values for the included SNPs in our study ranged from 15 to 367.

### Association of educational attainment with risk of infectious diseases

**Figure 1:**
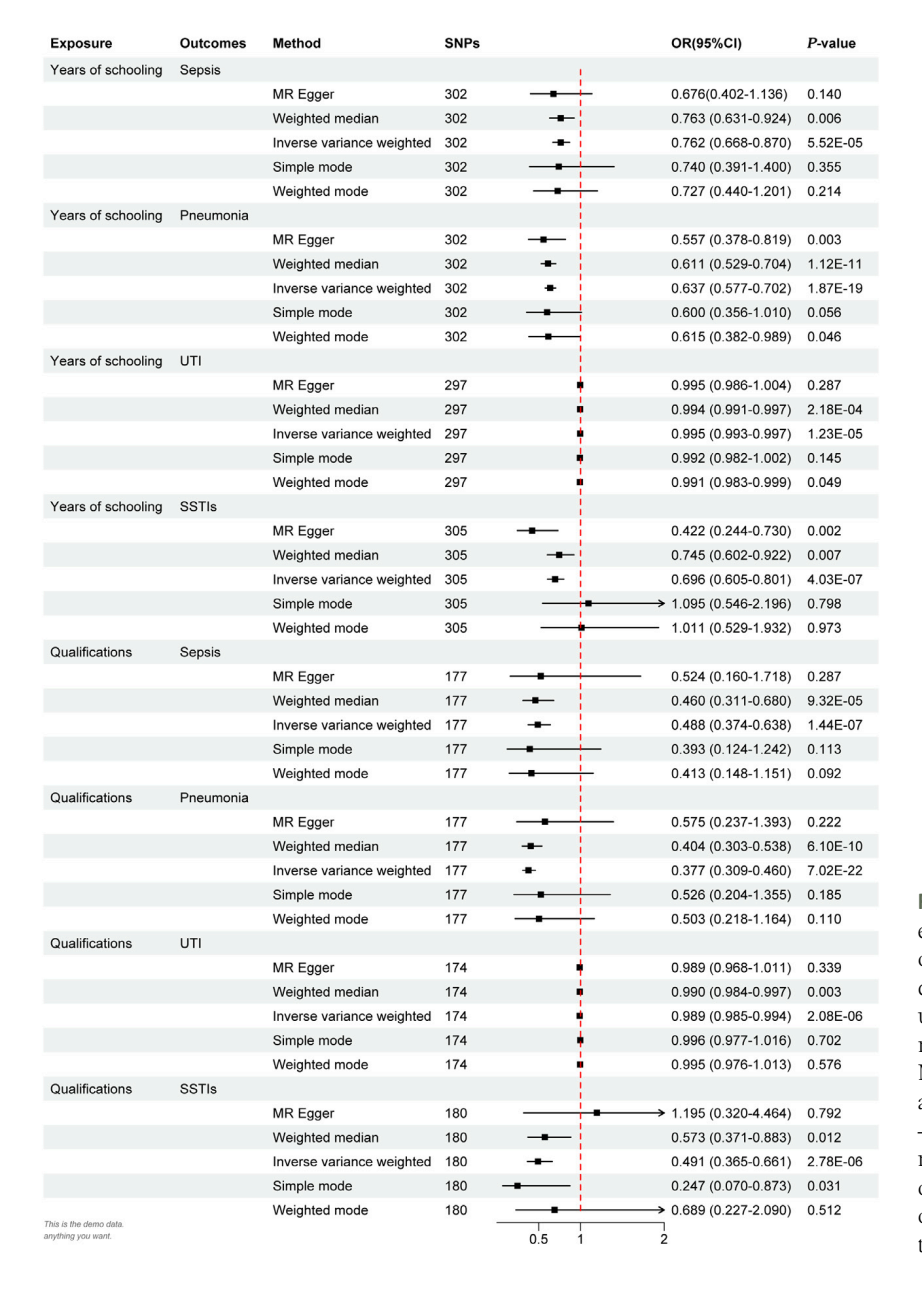
Univariable MR estimates for causal effects of years of schooling and qualifications (college or university degree) on the risk of infectious diseases. MR – Mendelian randomisation, OR – odds ratio, SNP – single-nucleotide polymorphism, SSTI – infection of the skin and subcutaneous tissue, UTI – urinary tract infection.

In the IVW estimates, increased years of schooling showed a significant association with a decreased risk of sepsis (odds ratio (OR) = 0.763; 95% confidence interval (CI) = 0.668–0.870, *P* = 5.525 × 10^−5^), pneumonia (OR = 0.637; 95% CI = 0.577–0.702, *P* = 1.875 × 10^−19^), UTIs (OR = 0.995; 95% CI = 0.993–0.997, *P* = 1.229 × 10^−5^), and SSTIs (OR = 0.696; 95% CI = 0.605–0.801, *P* = 4.034 × 10^−7^). We observed similar results regarding the correlation between qualifications and infectious diseases, where higher qualifications were causally associated with a greater decreased risk of sepsis (OR = 0.488; 95% CI = 0.374–0.638, *P* = 1.441 × 10^−7^), pneumonia (OR = 0.377; 95% CI = 0.389–0.460, *P* = 7.025 × 10^−22^), UTIs (OR = 0.989; 95% CI = 0.985–0.994, *P* = 2.085 × 10^−6^), and SSTIs (OR = 0.491; 95% CI = 0.365–0.661, *P* = 2.780 × 10^−6^) (**Figure 1**). The results of MR Steiger test indicated no reverse causal relationship between educational attainment and infectious diseases (Table S10 in the **Online Supplementary Document**).

### Multivariable Mendelian randomisation analyses

The multivariable MR analyses showed that years of schooling were associated with a decreased risk of sepsis (OR = 0.752; 95% CI = 0.659–0.859, *P* = 2 × 10^−5^), pneumonia (OR = 0.637; 95% CI = 0.576–0.704, *P* = 1 × 10^−18^), UTIs (OR = 0.996; 95% CI = 0.994–0.998, *P* = 1 × 10^−4^), and SSTIs (OR = 0.692; 95% CI = 0.560–0.798, *P* = 4 × 10^−7^) after adjusting for the weekly consumption of alcohol (Table S11 in the **Online Supplementary Document**). The findings remained consistent after adjusting for alcoholic drinks per week, smoking, and BMI. However, the multivariable MR analyses suggested there were no significant correlations between qualifications and SSTIs (OR = 0.727; 95% CI = 0.473–1.116, *P* = 0.145) after adjusting for BMI.

### Sensitivity analyses

**Figure 2:**
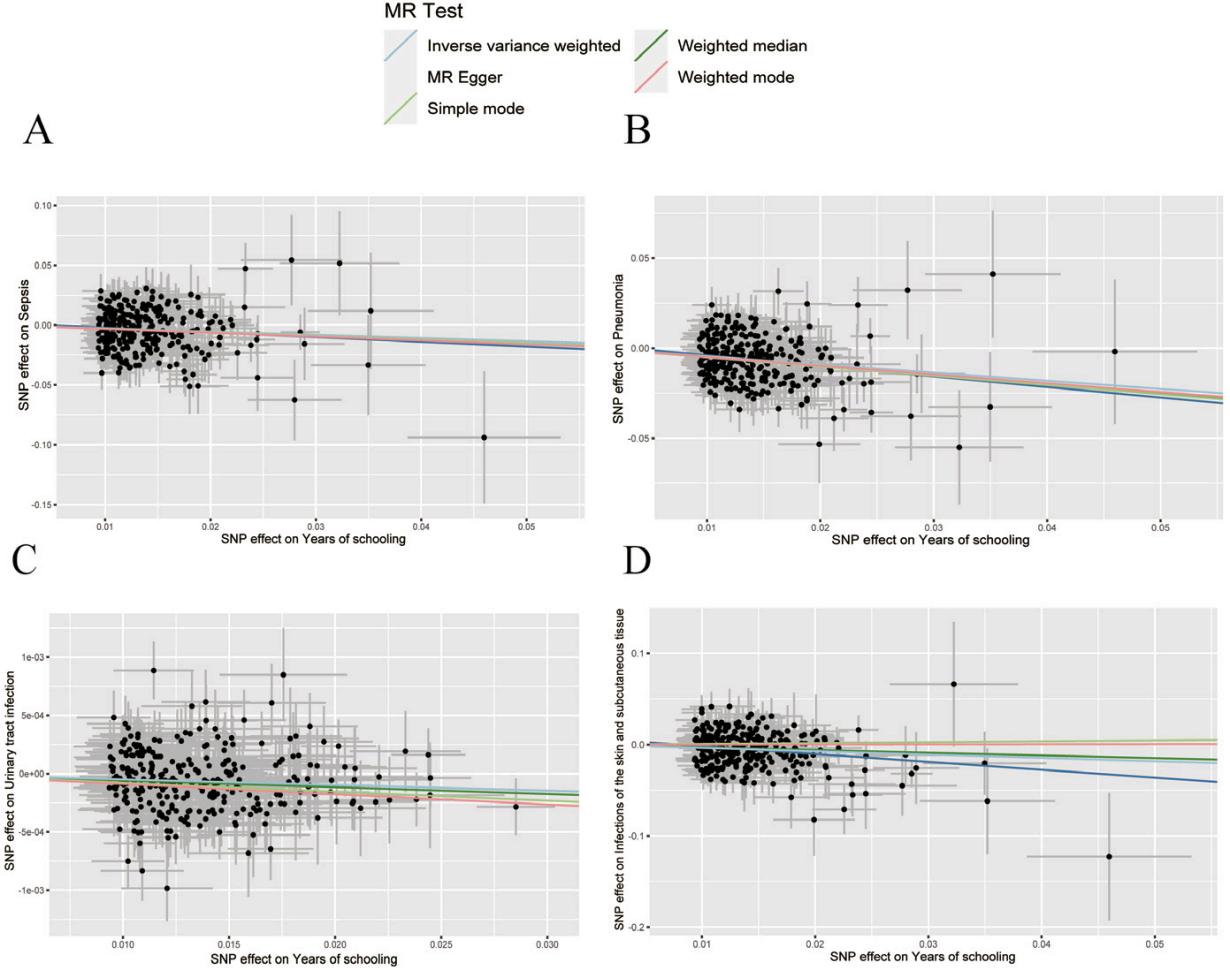
Scatter plot of the genetic risk of years of schooling. **Panel A.** Sepsis. **Panel B.** Pneumonia. **Panel C**. UTIs. **Panel D.** SSTIs. UTI – urinary tract infection, SSTI – infection of the skin and subcutaneous tissue.

**Figure 3:**
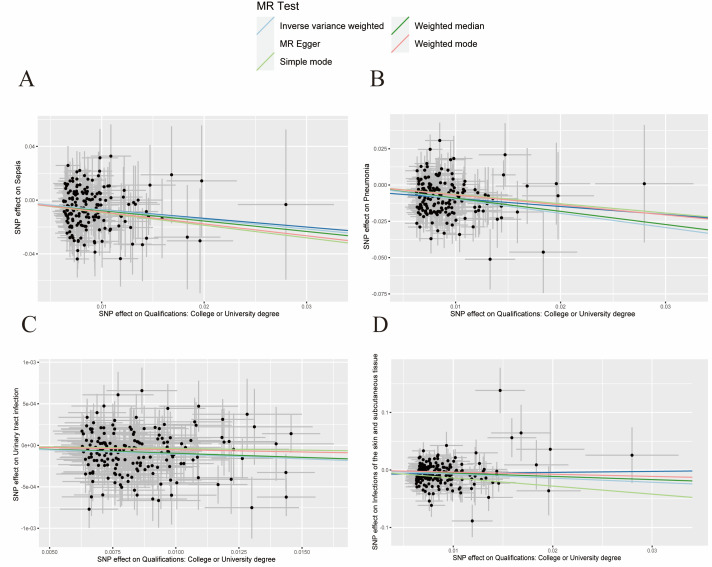
Scatter plot of the genetic risk of qualifications (college or university degree) **Panel A.** Sepsis. **Panel B.** Pneumonia. **Panel C.** UTIs. **Panel D.** SSTIs. UTI – urinary tract infection, SSTI – infection of the skin and subcutaneous tissue.

In the sensitivity analysis investigating the association between educational level and the risk of infectious diseases, we observerd no heterogeneity based on Cochran’s Q test (*P* > 0.05) (**Table 2**). There was also nos no evidence of horizontal pleiotropy between educational level and outcomes (*P* > 0.05), while the MR-PRESSO test did not identify any outlier SNPs. Meanwhile, the scatter plot showed a causal relationship between educational attainment and infectious diseases (**Figure 2**, **Figure 3**). The forest plot results also suggested a negative association between education level and the risk of infectious disease (Figure S1 in the **Online Supplementary Document**). while, the leave-one-out analysis showed that no single SNP significantly influenced the results, suggesting that the findings were robust (Figure S2 in the **Online Supplementary Document**).

**Table 2:** Evaluation of heterogeneity and pleiotropy using different methods

	Heterogeneity		
**Outcomes by exposure**	**MR-Egger *P*-value**	**IVW *P*-value**	**Pleiotropy *P*-value**
Years of schooling			
*Sepsis*	0.278	0.289	0.638
*Pneumonia*	0.109	0.113	0.481
*UTI*	0.387	0.403	0.988
*SSTIs*	0.648	0.609	0.065
Qualifications			
*Sepsis*	0.915	0.924	0.895
*Pneumonia*	0.287	0.288	0.339
*UTI*	0.381	0.402	0.895
*SSTIs*	0.282	0.267	0.178

IVW – Inverse-variance weight, MR – Mendelian randomisation, SSTI – infections of the skin and subcutaneous tissue, UTI – urinary tract infection

## DISCUSSION

In this MR study, we used large-scale GWAS summary data to investigate the causal association between educational level and the risk of infectious diseases, encompassing sepsis, pneumonia, UTIs, and SSTIs among individuals of European ancestry. Our findings indicate that a high educational level may be causally associated with a decreased risk of infectious diseases.

Education serves as a crucial indicator of socioeconomic status and plays a significant role in social inequality [11,35]. In recent years, there has been an increasing interest in observational studies examining the association between educational levels and the occurrence and progression of various infectious diseases. In an observational study involving 65 227 participants, individuals with lower educational levels were found to have a hazard ratio of 1.28 (95% CI = 1.13–1.46) for sepsis compared with those with higher educational levels [36]. Furthermore, a trend analysis of 86 677 death records in Colombia spanning the period from 1998 to 2005 showed a significantly higher pneumonia mortality rate among adults aged 25 and above with lower educational levels [13]. Additionally, a cross-sectional analysis involving 41 869 women found that individuals with some high school education had a prevalence ratio of 2.06 (95% CI = 1.77–2.40) for UTIs compared to those with graduate school education [14]. However, it is important to acknowledge that these observational studies are susceptible to confounding factors and reverse causality, thereby limiting their capacity to definitively establish causal relationships.

Moreover, few studies have investigated the association between educational level and SSTIs. To address this gap, we used the MR method to investigate the causal relationship between education level and four infectious diseases (sepsis, pneumonia, UTIs, and SSTIs). The univariable MR results suggest that there may be a causal relationship between increased education level and decreased risk of infectious diseases. Specifically, the educational level had the greatest effect on SSTIs, while its influence on UTIs was minimal. Further research should explore the underlying mechanisms behind these disparities.

Previous studies have established a significant correlation between smoking, alcohol consumption, higher BMI, and increased incidence of infectious diseases [19,29,30,37]. To account for the potential influence of these confounding factors on our results, we conducted multivariable MR analyses and found that most results remained consistent with the findings of our single-factor analysis, further confirming their robustness. Our comprehensive analyses based on both univariable and multivariable MR approaches suggest that policymakers should consider educational attainment when assessing infectious disease risk. They should also be aware of its potential role in improving public health, where investments in education should be encouraged when making public health policy. Promoting primary education and enhancing accessibility to educational resources may mitigate the occurrence of infectious diseases, while community-based health education initiatives can bolster public awareness of infectious diseases. Encouraging lifelong learning at the individual level could also help in improving health outcomes.

In line with previous studies, our results indicate a causal correlation between lower educational attainment and heightened vulnerability to infectious diseases. Notably, individuals with higher educational levels were found to have superior health care self-management and engagement practices [38]. Moreover, studies have suggested that a high level of education is significantly associated with a decreased prevalence of smoking, obesity, and heavy alcohol consumption, as well as enhanced cognitive abilities for accessing accurate health care information [39,40]. Furthermore, education was found to have a key role in determining one's social and economic status [35,41], whereby individuals with higher education typically have higher income and occupational status; this, in turn, allows them access to a healthier living environment, timely medical care, and treatments for infectious [42]. For example, children from middle to high-income families were reported to have lower blood lead levels [43], while those from socioeconomically disadvantaged backgrounds were found to be more susceptible to exposure to infectious pathogens [44]. Further research should attempt to explore the existence of other mechanisms in this relationship.

Our study had two sets of genetic instruments related to educational levels, where we still observed consistent causality between educational attainment and the risk of four specific infectious diseases. However, we have to acknowledge several limitations. First, the use of GWAS data primarily from European populations limits the generalisability of our findings to other ethnic groups due to the association of genetic variants with birth location [45], suggesting a need for further research in other contexts. Nonetheless, our exposure and outcome data sources demonstrated racial consistency, thereby mitigating potential biases arising from diverse genetic backgrounds. Second, previous MR studies have demonstrated that smoking, drinking, and BMI influence infectious diseases. Although we attempted to account for the these confounding factors through our multivariable MR analysis, other unidentified confounding factors might exist, such as health care access, environmental factors, and others. Thus, the causality of this relationship should be considered with caution, and these factors should be explored in future research. Finally, our study solely provided genetic-level evidence by using pre-existing GWAS data sets. Because the original data collection methods and purposes might influence the results, additional further research is necessary to validate this potential causal relationship.

## CONCLUSIONS

This MR study provided evidence that high educational attainment may be associated with a decreased risk of sepsis, pneumonia, UTIs, and SSTIs, suggesting it might be a protective factor against the risk of infectious diseases.

## Additional material


Online Supplementary Document
