# Supplementary figures and images for: A causal relationship between educational attainment and risk of infectious diseases: A Mendelian randomisation study
Source: J Glob Health. 2024 Apr 26;14:04089. doi: 10.7189/jogh.14.04089 (PMC11046428; doi:10.7189/jogh.14.04089)

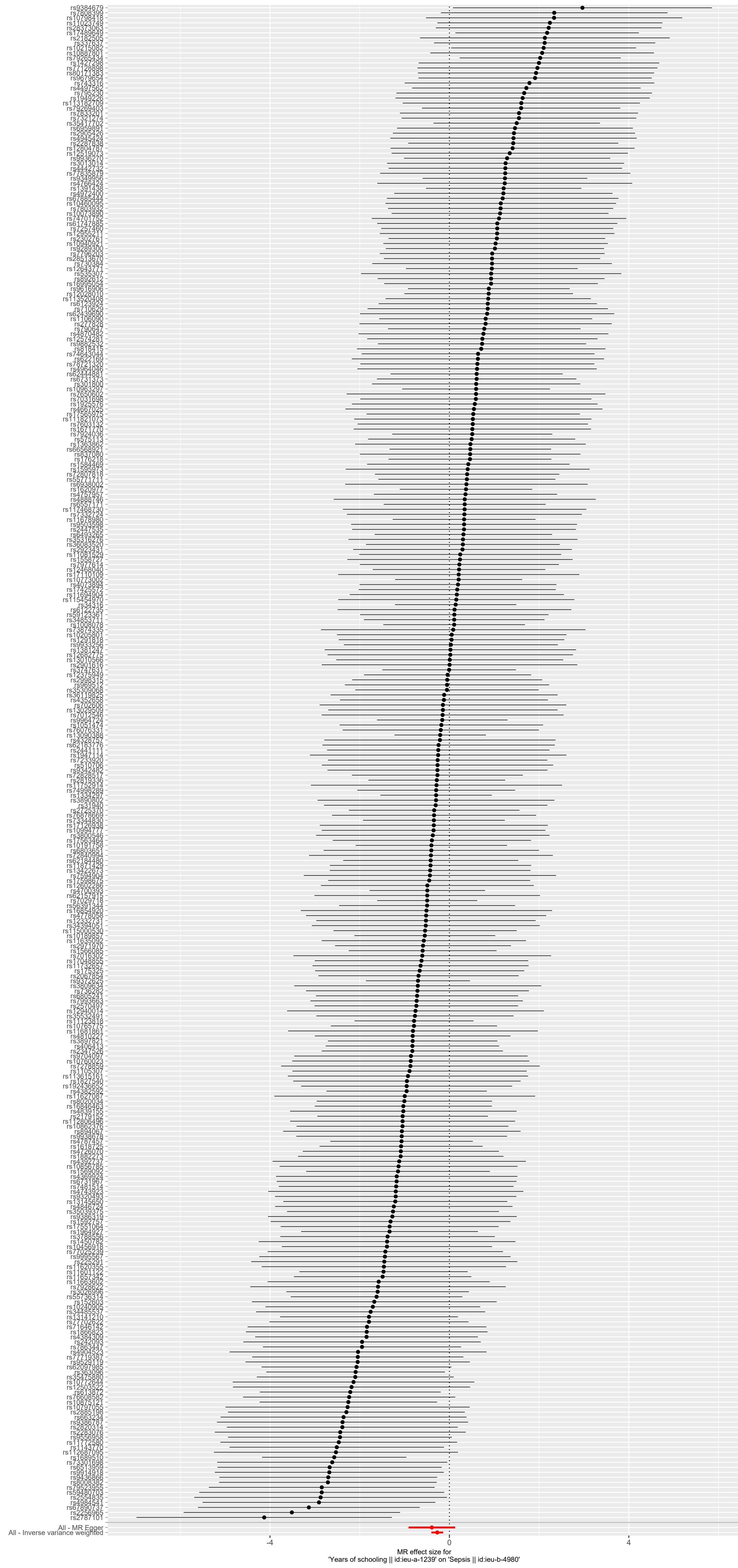

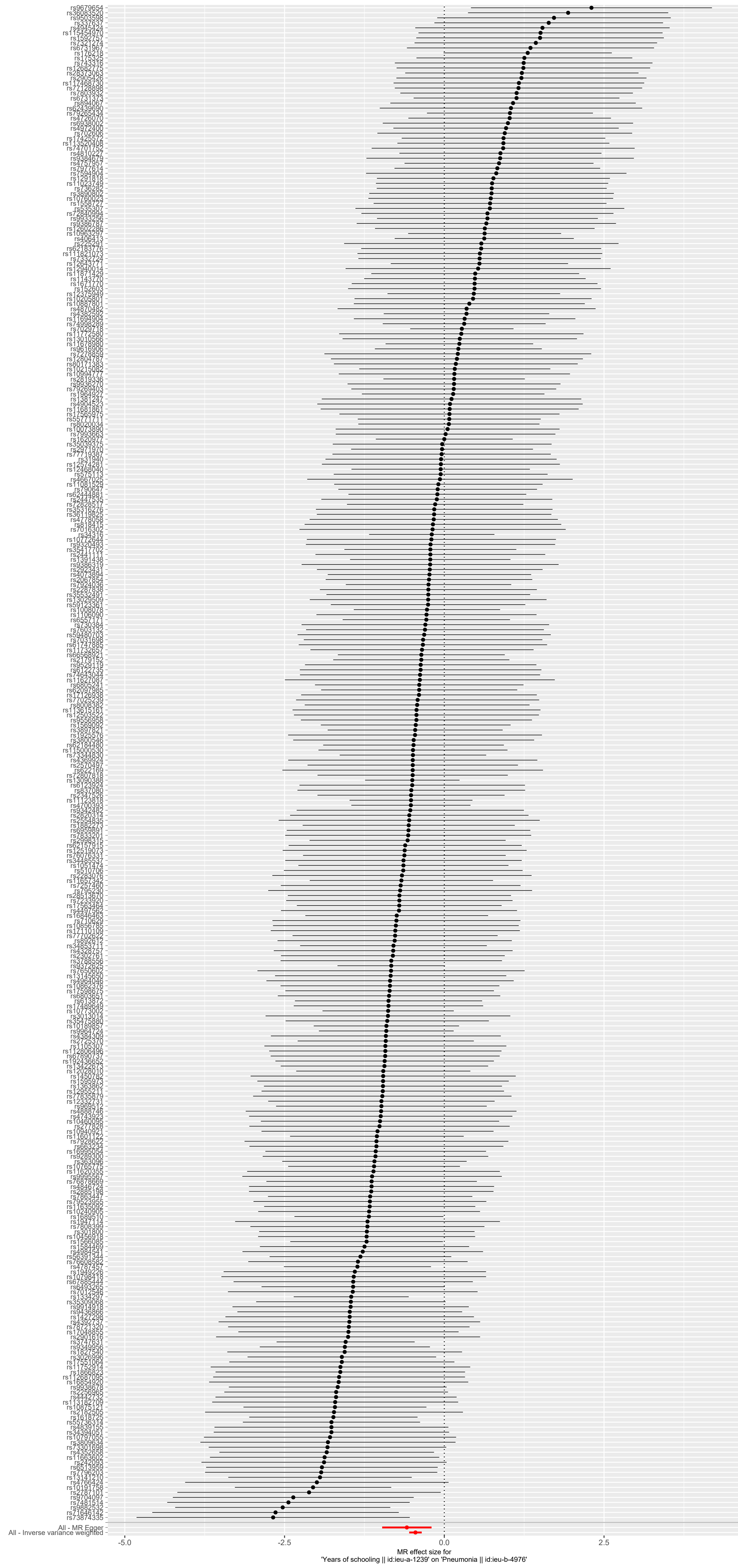

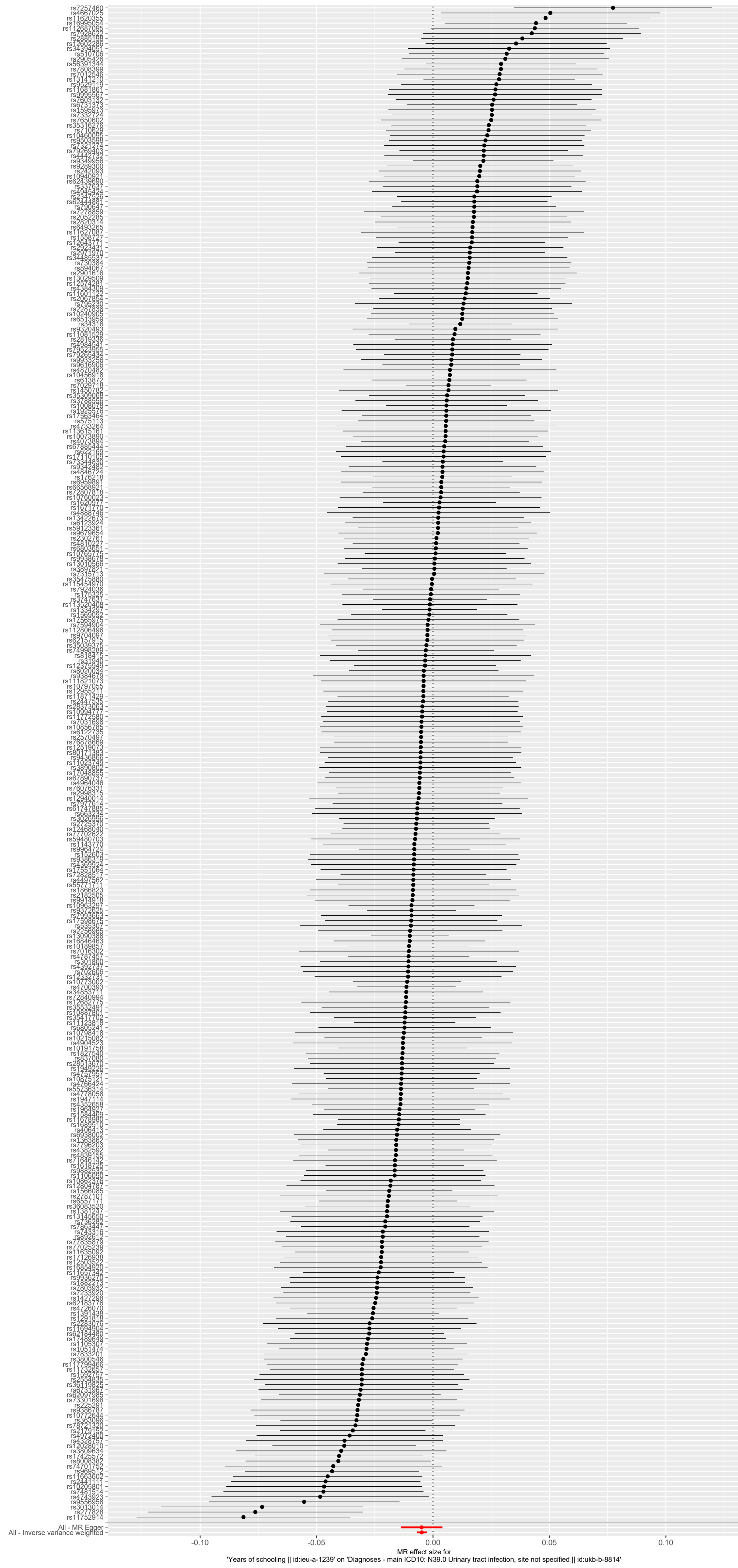

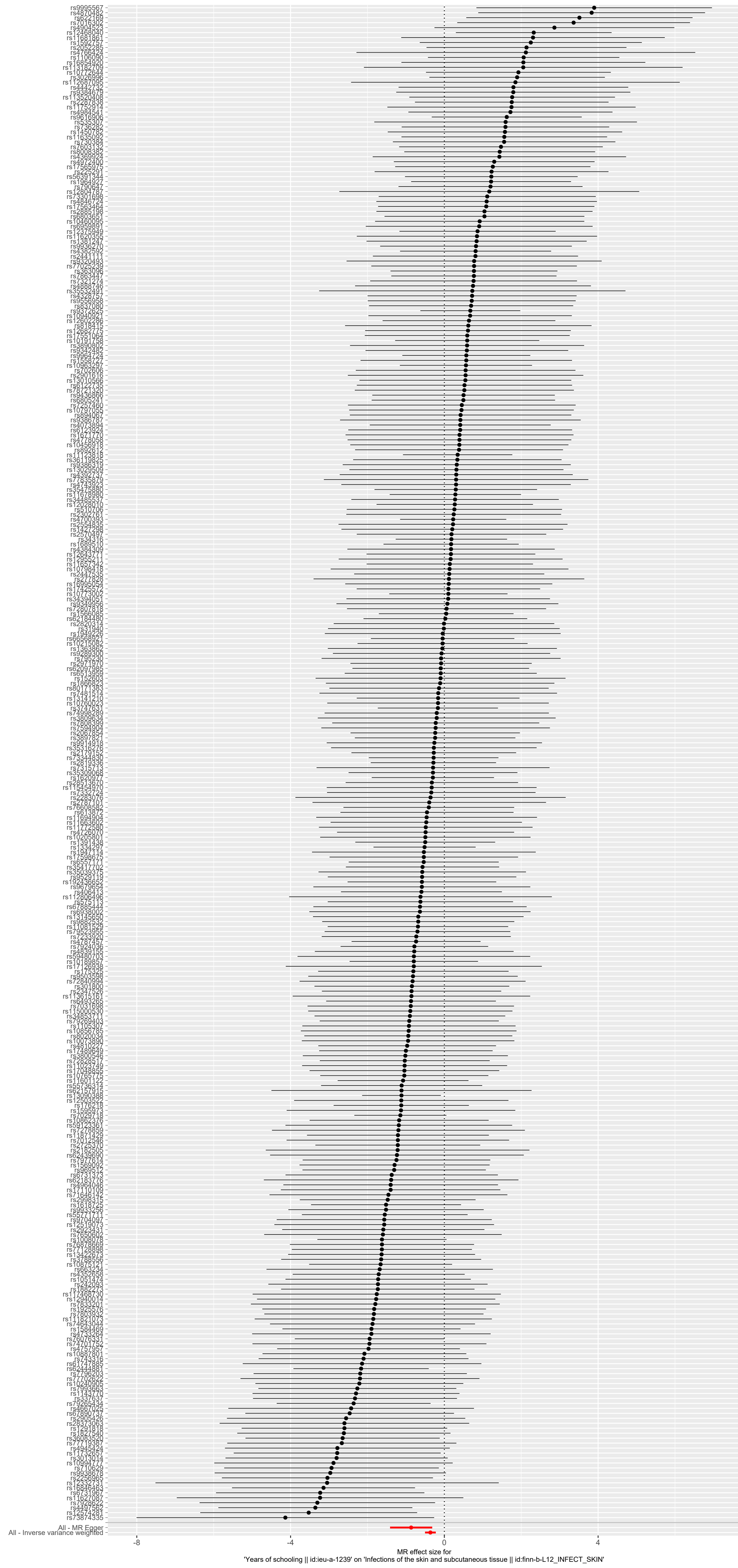

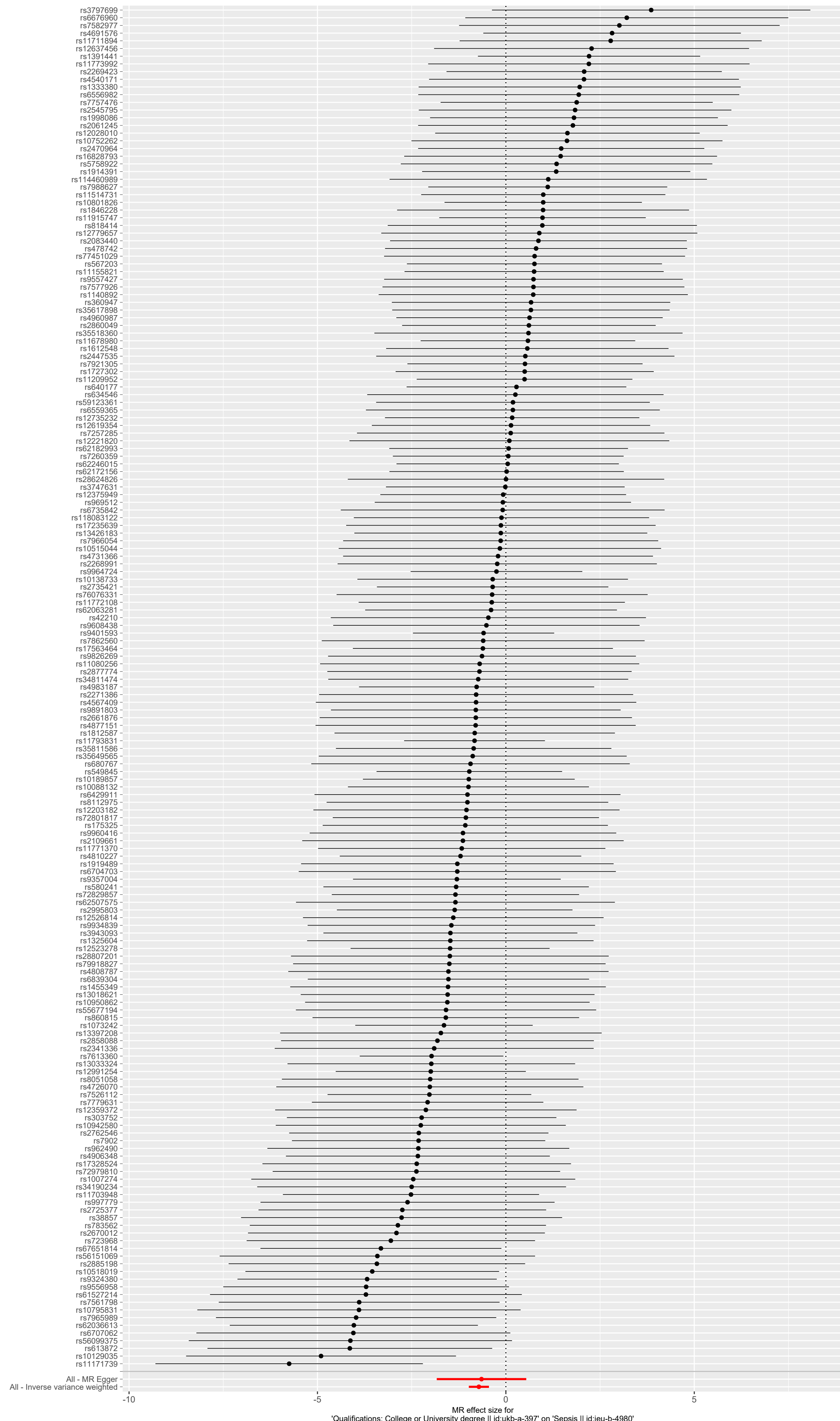

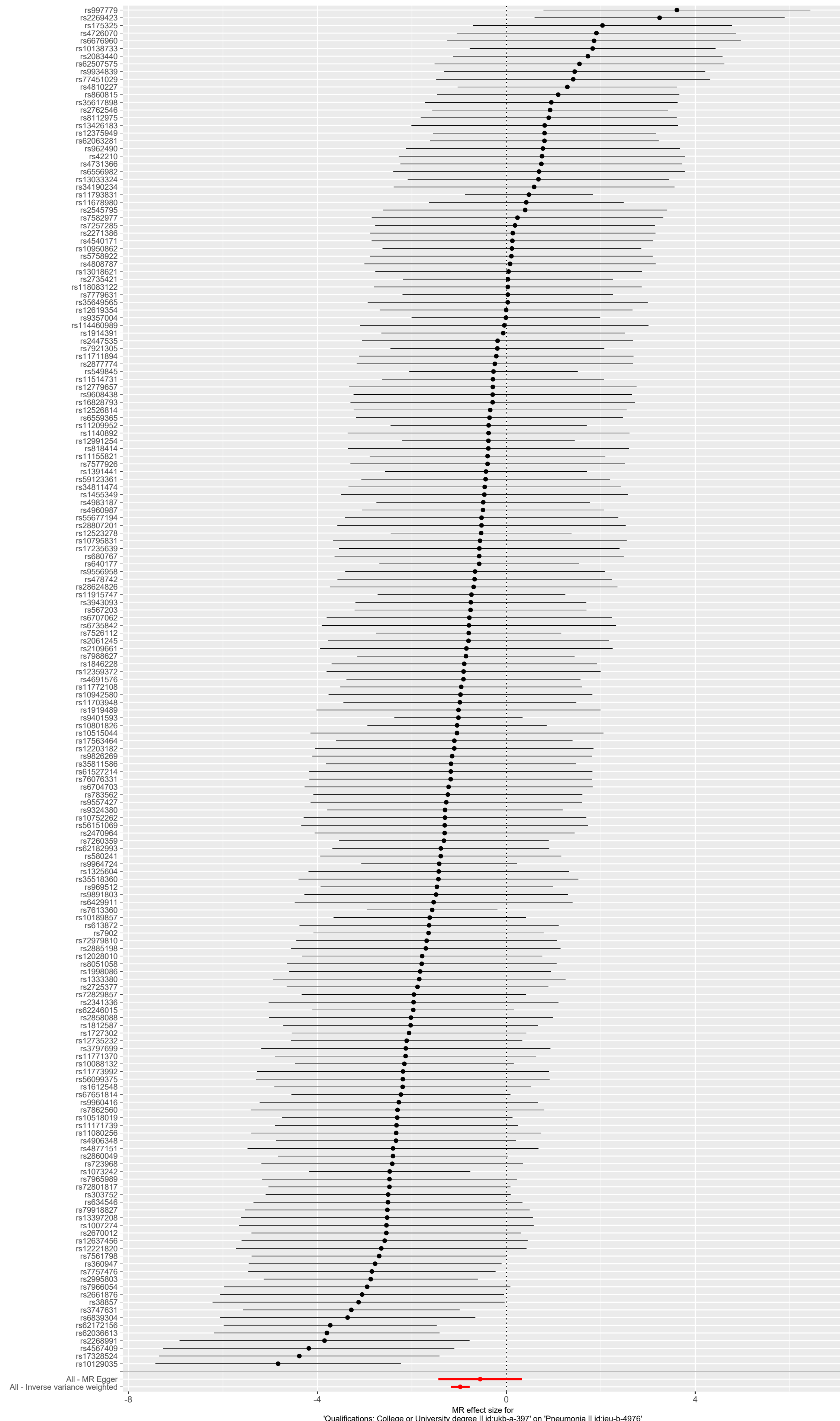

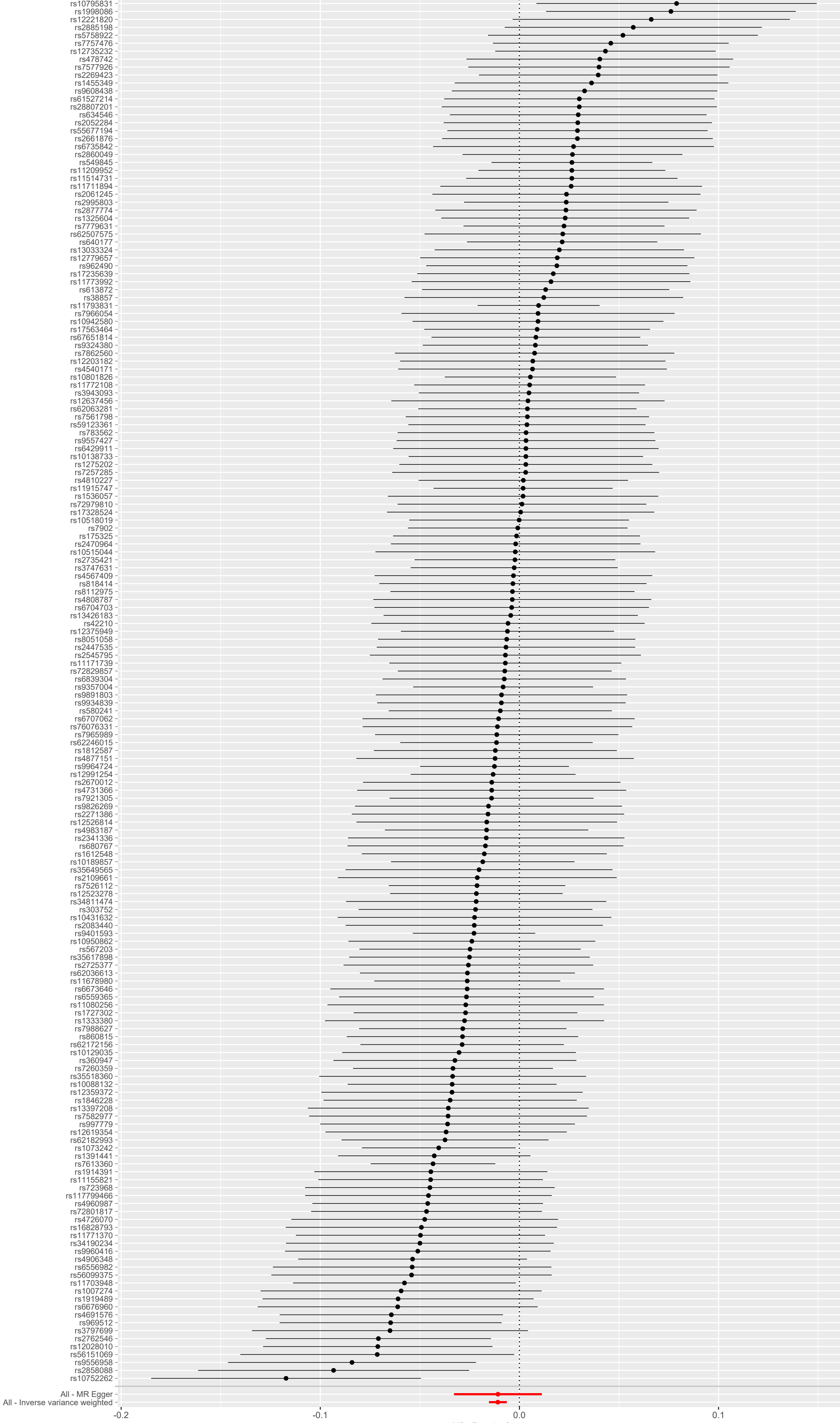

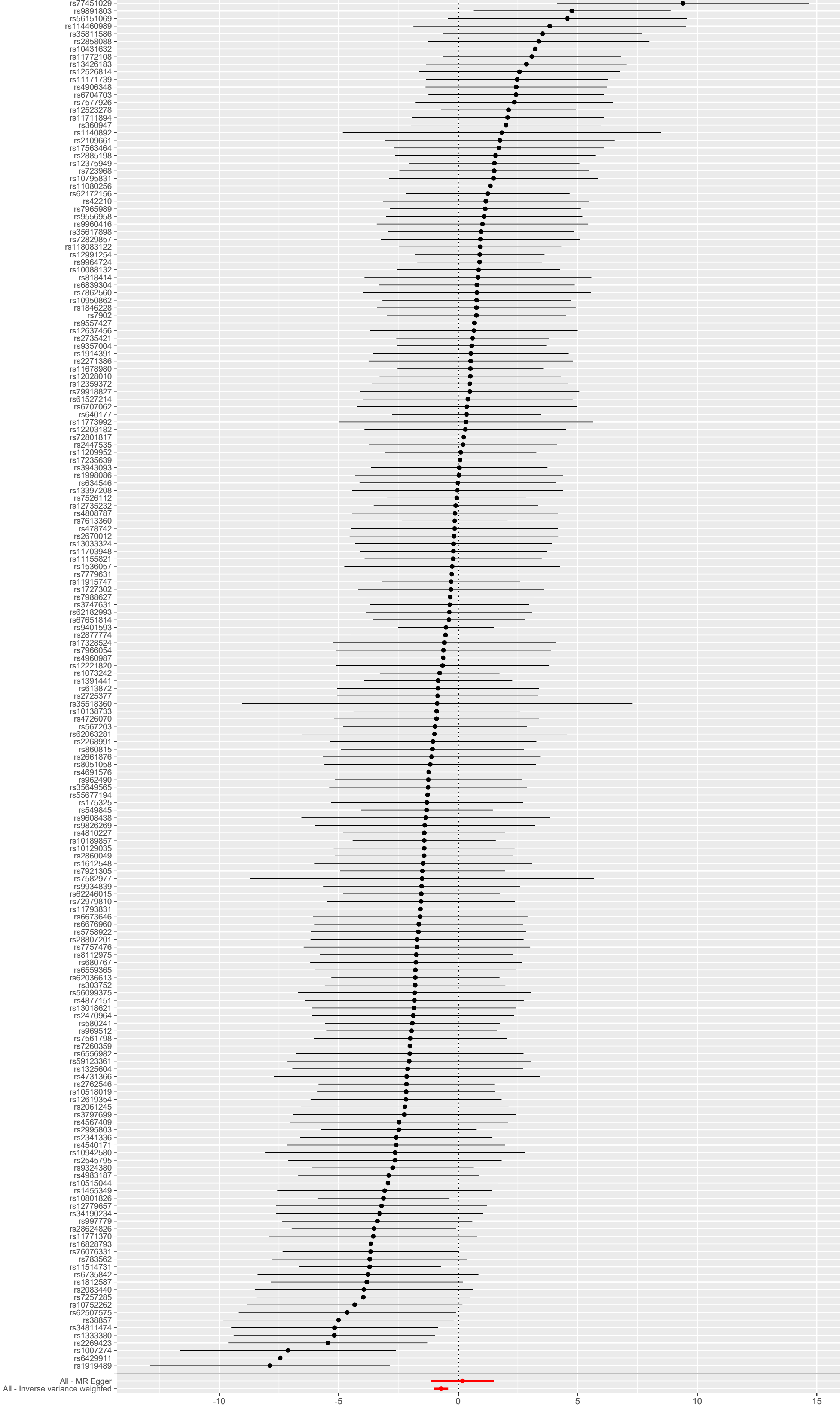

Supplement: Online Supplementary Document [file jogh-14-04089-s001.zip › jogh-14-04089-SF1.pdf]

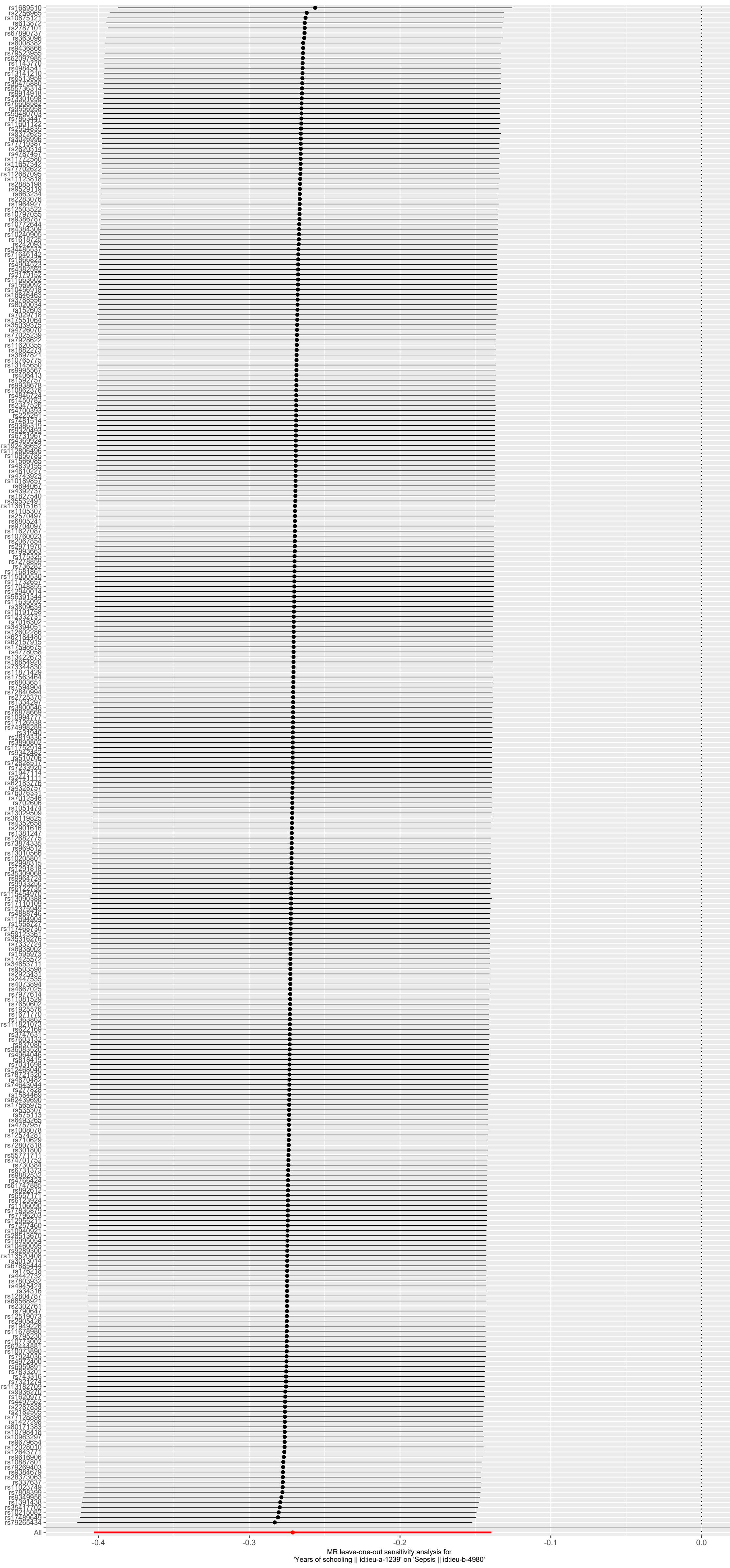

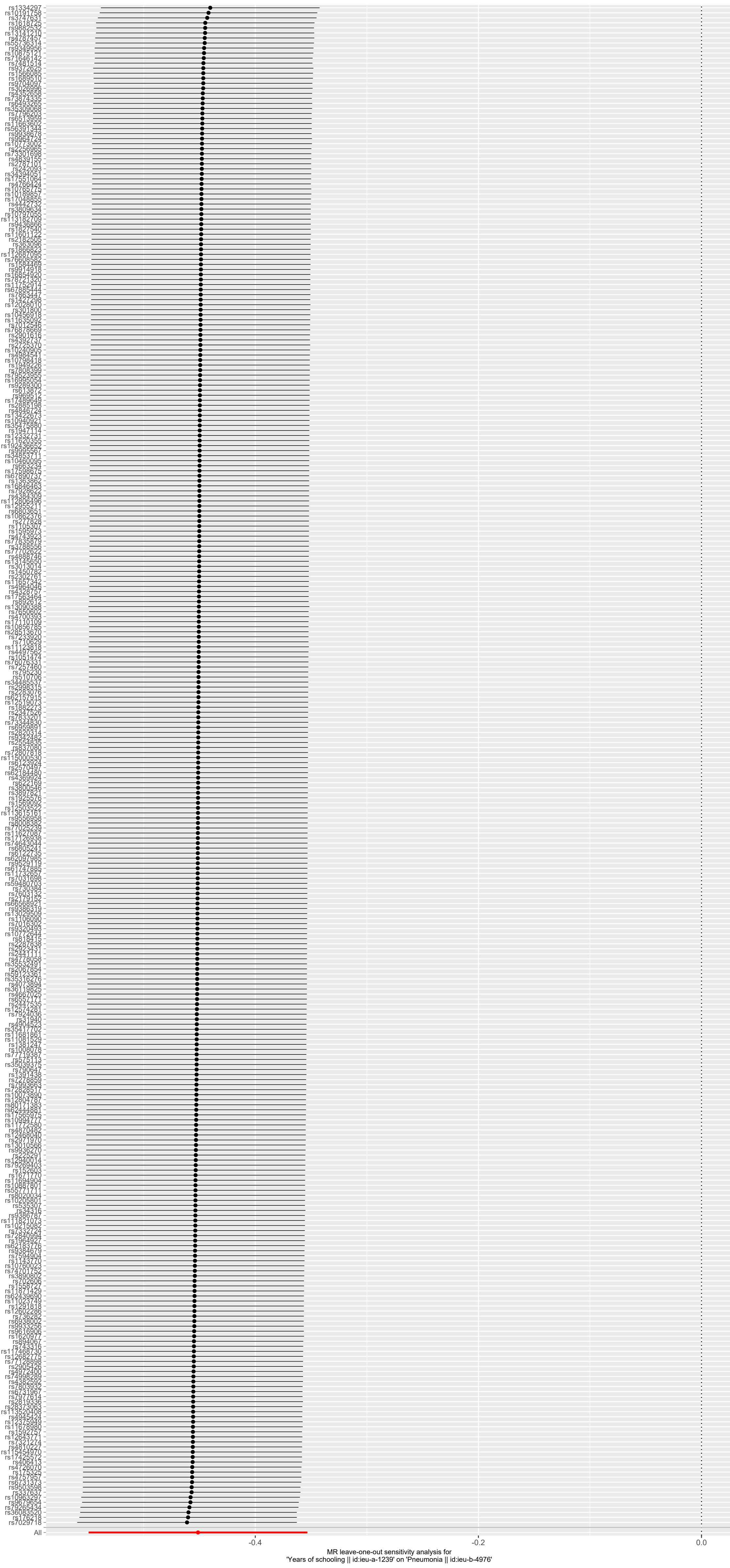

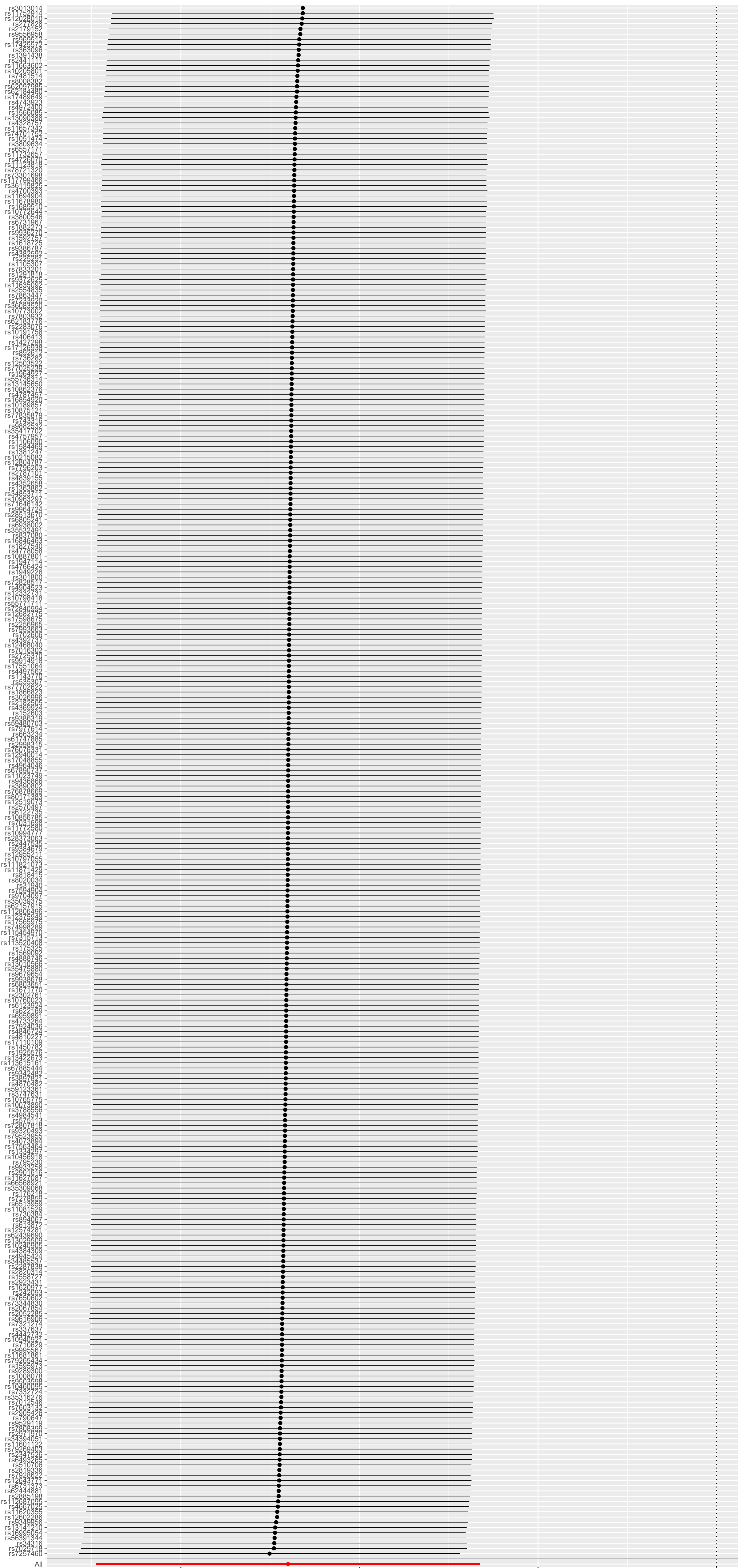



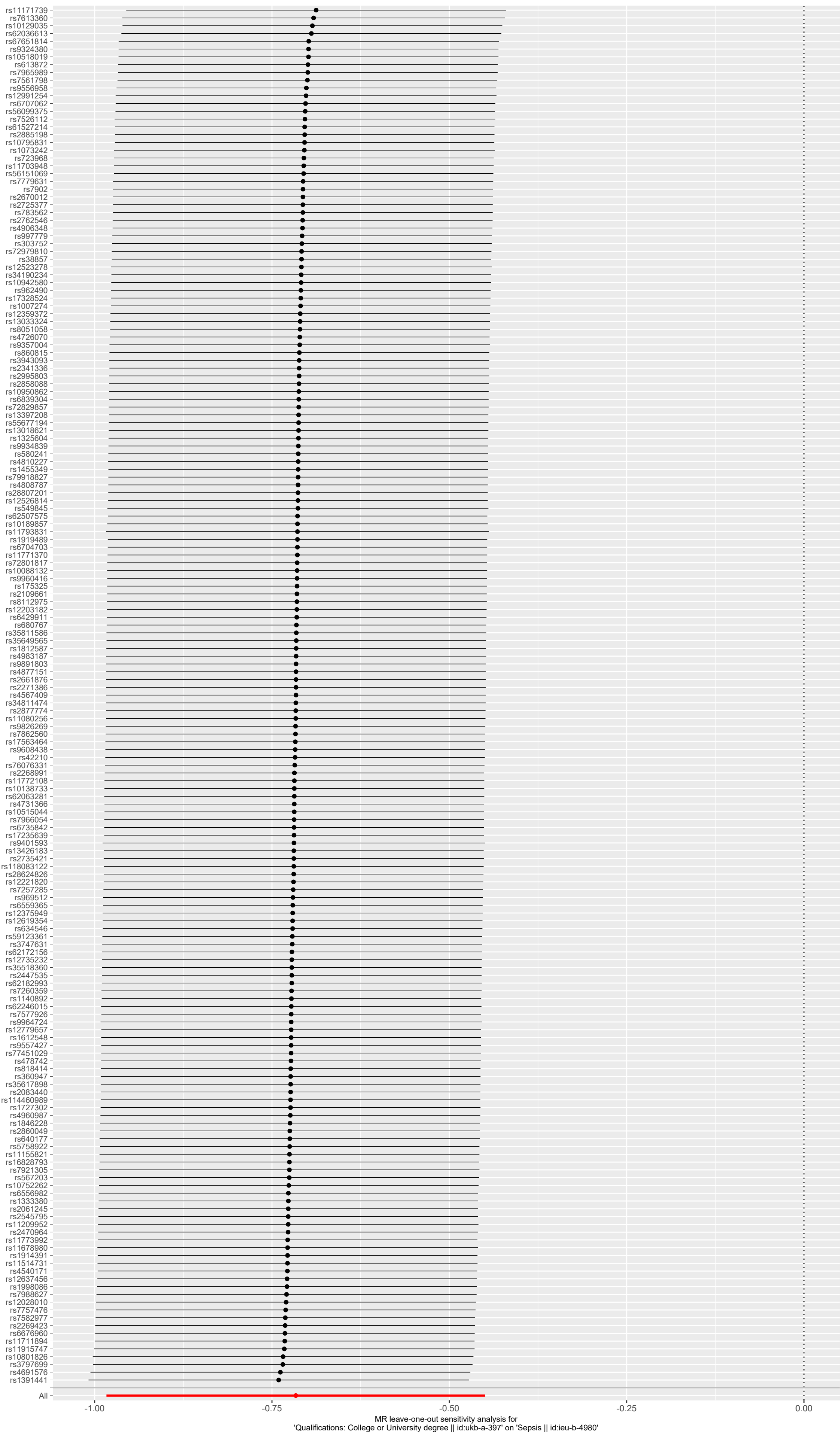

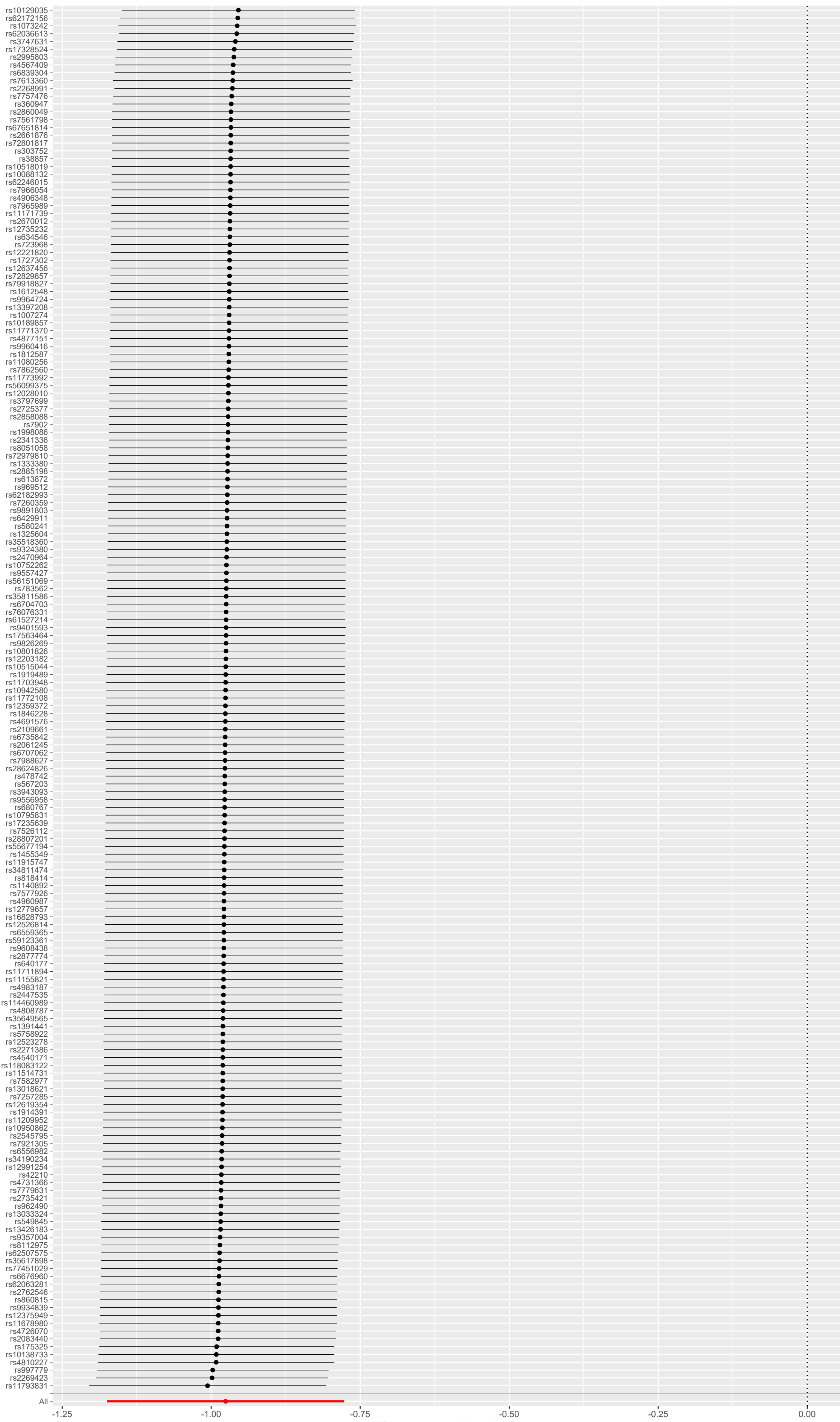

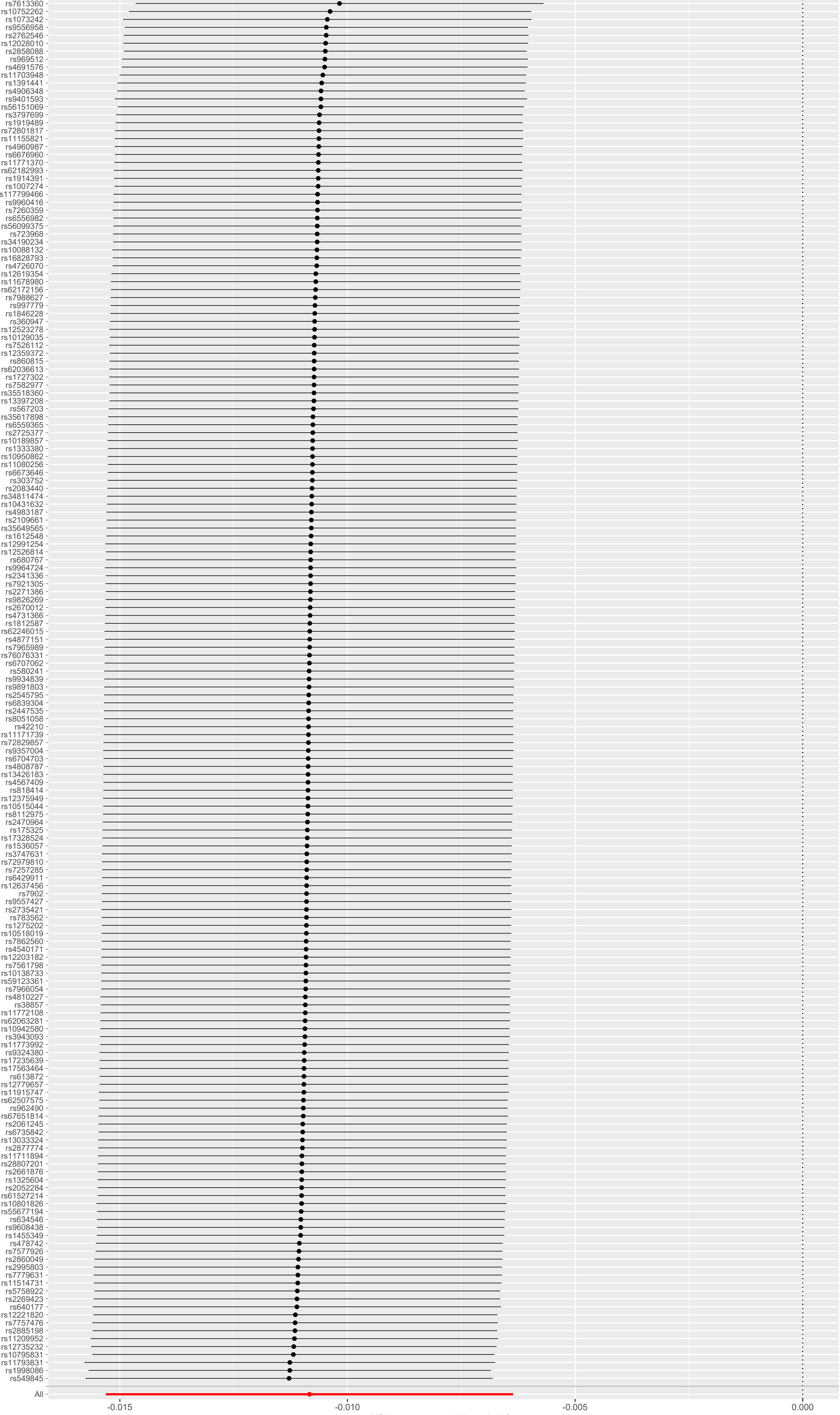

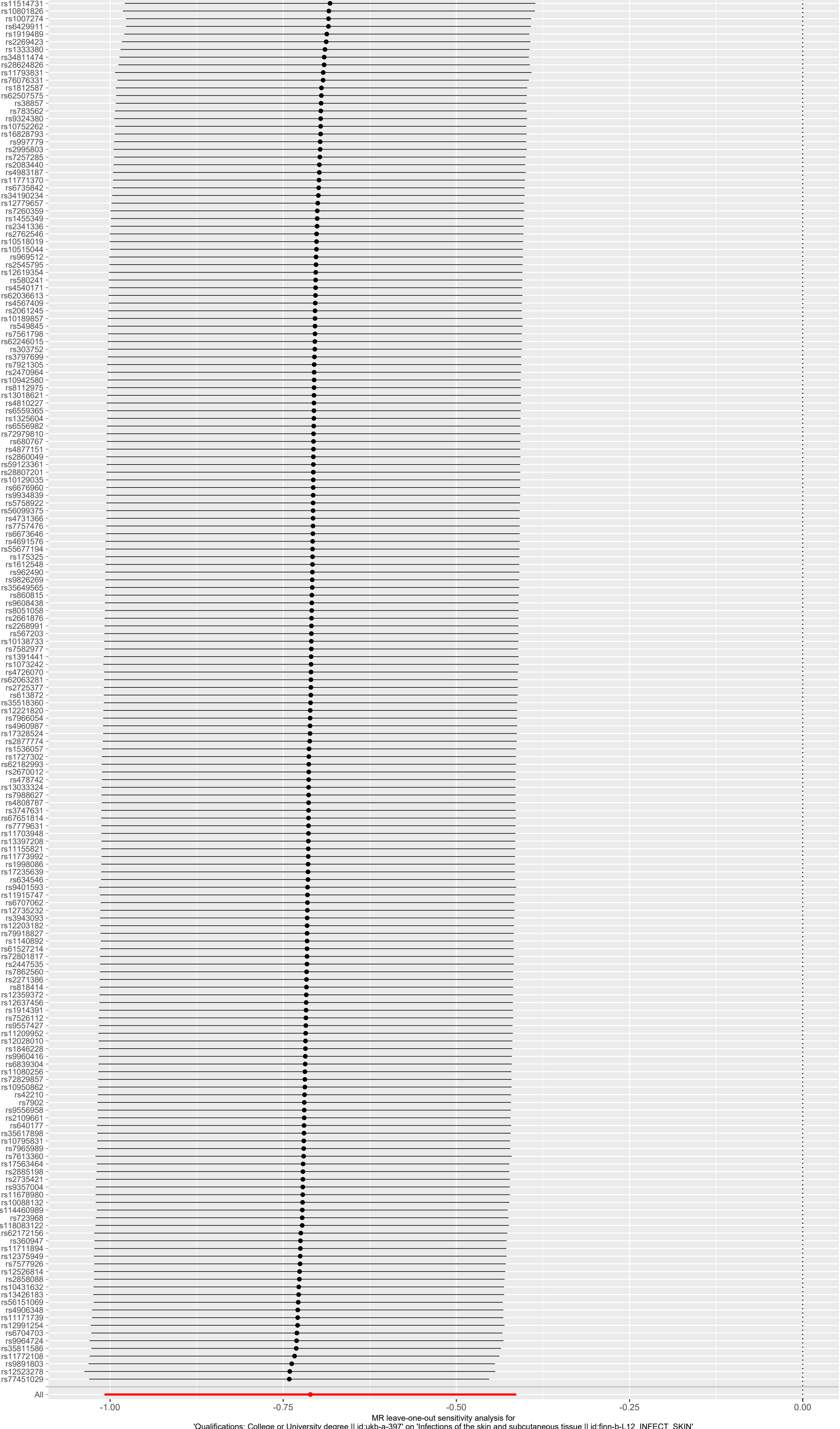

Supplement: Online Supplementary Document [file jogh-14-04089-s001.zip › jogh-14-04089-SF2.pdf]
